# Meaningful engagement: A crossfunctional framework for digital therapeutics

**DOI:** 10.3389/fdgth.2022.890081

**Published:** 2022-08-11

**Authors:** Gabriel Strauss, Jessica E. Flannery, Elise Vierra, Xin Koepsell, Emily Berglund, Ian Miller, Jessica I. Lake

**Affiliations:** Limbix Health, San Francisco, California, United States

**Keywords:** digital therapeutics, engagement, meaningful engagement, clinical outcomes, adolescence

## Abstract

Digital mental health interventions, or digital therapeutics, have the potential to transform the field of mental health. They provide the opportunity for increased accessibility, reduced stigma, and daily integration with patient's lives. However, as the burgeoning field continues to expand, there is a growing concern regarding the level and type of engagement users have with these technologies. Unlike many traditional technology products that have optimized their user experience to maximize the amount of time users spend within the product, such engagement within a digital therapeutic is not sufficient if users are not experiencing an improvement in clinical outcomes. In fact, a primary challenge within digital therapeutics is user engagement. Digital therapeutics are only effective if users sufficiently engage with them and, we argue, only if users meaningfully engage with the product. Therefore, we propose a 4-step framework to assess meaningful engagement within digital therapeutics: (1) Define the measure of value (2) Operationalize meaningful engagement for your digital therapeutic (3) Implement solutions to increase meaningful engagement (4) Iteratively evaluate the solution's impact on meaningful engagement and clinical outcomes. We provide recommendations to the common challenges associated with each step. We specifically emphasize a cross-functional approach to assessing meaningful engagement and use an adolescent-focused example throughout to further highlight developmental considerations one should consider depending on their target users.

## Introduction

Digital therapeutics are evidence-based mental health therapeutic interventions driven by software programs to prevent, manage, or treat a medical disorder or disease (1). The demand for digital therapeutics is steadily increasing as the demand for mental health services increases, but existing face-to-face services remain limited. Digital therapeutics have the opportunity to transform the field of mental health– improving access to quality mental health services and the mental health of populations previously neglected in treatment options. Several factors can further limit an individual's ability or desire to seek face-to-face treatment, such as stigma, cultural acceptance, embarrassment, access to services, financial constraints, or a preference for self-reliance (2, 3). For developmental populations, access can be further limited by transportation, child-care for other siblings, or caregiver alignment with the need for therapy (2, 3). While digital therapeutics offer a promising solution to barriers in access to care, user engagement within digital therapeutics is a primary challenge the industry faces (4). Without sufficient user engagement, the success and promise of digital therapeutics is limited.

**Figure 1:**
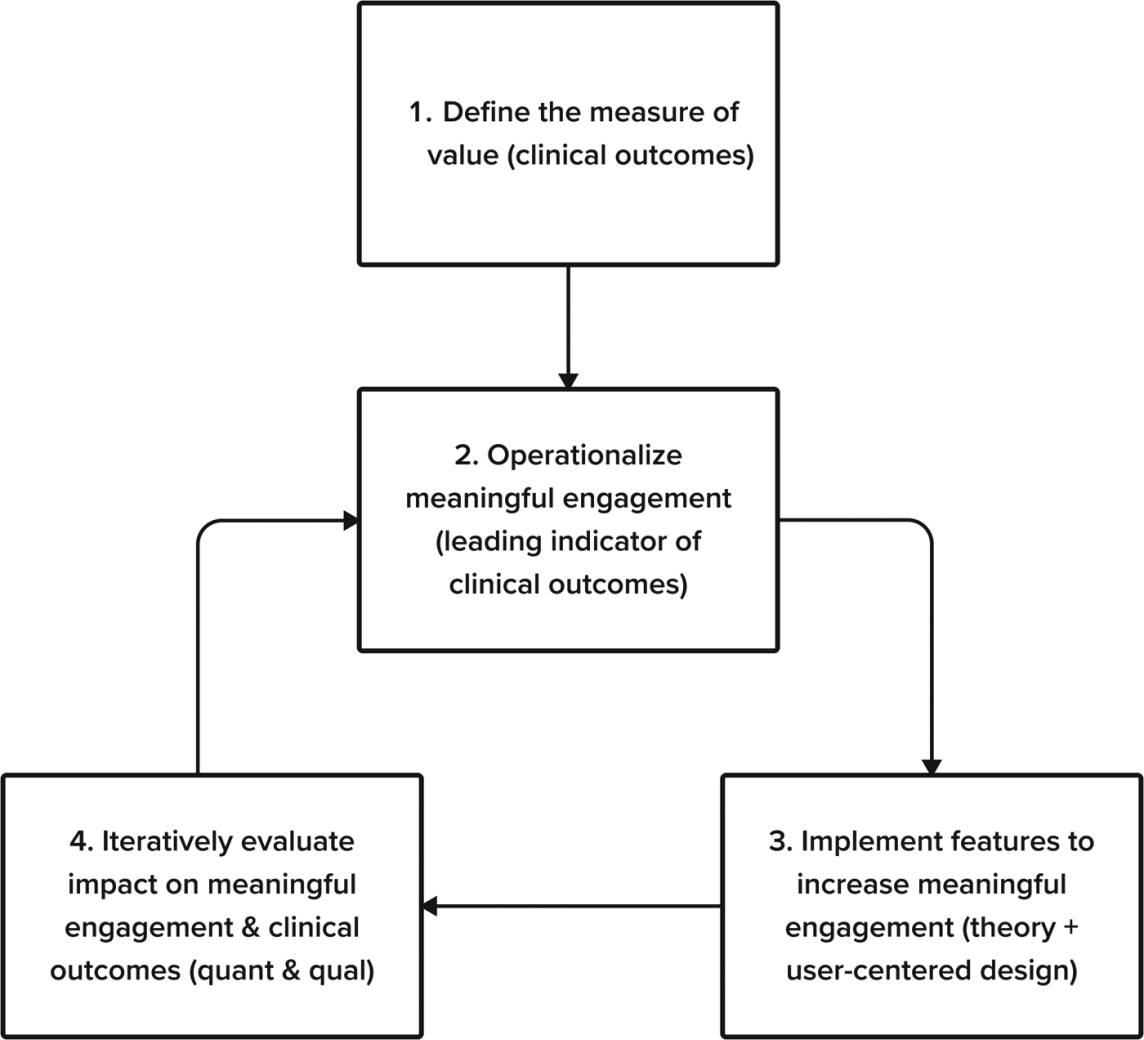
Our 4-step framework to address common challenges and recommendations for identifying, measuring, and driving meaningful engagement in digital therapeutics.

To encourage sufficient engagement, we believe it's critical to integrate cross-functional perspectives from clinical science, product management, product design, content, and user experience research teams to assess common challenges and recommendations for engagement within digital therapeutics. Within this context, we discuss the various definitions of engagement and advocate for alignment with a focus on *measures of meaningful engagement.* Drawing from work in the consumer and software as a service (SaaS) industries, we propose a 4-step framework to address common challenges and recommendations for identifying, measuring, and driving meaningful engagement in digital therapeutics: (1) Define the measure of value (2) Operationalize meaningful engagement for your digital therapeutic (3) Implement solutions to increase meaningful engagement (4) Iteratively evaluate the solution's impact on meaningful engagement and clinical outcomes (See Figure 1.) This process uses the build-measure-learn cycle, emphasizing theory, user-centered design, and quantitative and qualitative feedback. We focus on implementing our four-step process in digital therapeutic development targeting adolescents. We also provide recommendations to overcome the general challenges digital therapeutics face in assessing and encouraging meaningful engagement in treatment within and outside of the app.

## Defining engagement

While there is broad agreement that product engagement is critical for digital therapeutics to be impactful, the precise definition of engagement and measurement processes are vague (5–7). Increasingly, digital health technologists and researchers agree that assessing “generic” measures of engagement (e.g., number of sessions, weekly active usage, or program completion) may not be sufficient if they are not strong mediators of outcomes (8–10). Consequently, there is a growing call to follow a clinically informed and data-driven approach to identify specific engagement metrics that uniquely predict the long-term value for a digital therapeutic, referred to as *measures of meaningful engagement* (4, 9, 11, 12).

## Driving meaningful engagement

To drive meaningful engagement in a digital therapeutic we built upon two well-known frameworks by adapting them for the unique challenges of digital therapeutic development: The Build-Measure-Learn framework popularized by The Lean Startup and the Design Thinking framework popularized by IDEO (13, 14). Both processes have significant similarities (15). They each involve building prototypes, testing and measuring the success of those prototypes through qualitative user feedback and quantitative experiment, generating insights and applying them to subsequent iterations of prototyping, testing, and learning. A key tenet of the Lean Startup framework is to define an appropriate metric for the success of a product (13). As has already been mentioned, the digital health industry needs to move away from generic measures of engagement, and identify metrics that predict clinical outcomes. We therefore elevated this into dedicated steps in our process. A key tenet of design thinking is to “understand” and “observe” users through the synthesis of existing research and by directly engaging with users as part of the design process (14). In line with this, our process emphasizes drawing upon clinical science and theory, and involving users as active partners in the design process.

We distilled these elements into a 4-step process-oriented framework for driving meaningful engagement in digital therapeutics that accounts for the unique challenges faced by developers of digital therapeutics.

## 1: Define the measure of value

Defining the measure of value is an area in which there is a fundamental difference between digital therapeutics and most consumer or SaaS products. For example, in a consumer product like TikTok, users are looking to be entertained, and therefore retention (consistently coming back to the app) is an excellent measure of the value of that product. Alternatively, most digital therapeutics' primary measure of value is clinical outcomes. Users have health needs and digital therapeutics must address those needs. If users come back to the app every day for months (strong retention), but their symptoms do not improve, they have not received the primary intended value from the product.

**Recommendation: Optimize for clinical outcomes.** As outlined above, for most digital therapeutics, the primary goal is to improve patients' clinical outcomes. Take this example of a cognitive behavioral therapy (CBT)-based digital therapeutic for adolescent depression. Patients and their parents, providers, and payors all care about improving patients' depressive symptoms. We analyze the decrease in the patient health questionnaire score (PHQ score; a measure of depressive symptom severity; (16). As such, we evaluate all efforts to improve engagement against their impact on reducing PHQ scores. We continue with this example below.

## 2: Operationalize meaningful engagement for your digital therapeutic

After identifying the measure of value, a common challenge is selecting the engagement behaviors that are the strongest leading indicators of that value. In product development, there are often limited resources; therefore, efforts must be focused where they will have the greatest clinical return on investment.

In our example digital therapeutic, we seek the *best* leading indicator of a drop in PHQ score. Such engagement metrics usually correspond to interactions with the “active ingredients” in a digital therapeutic. Just as traditional medicine can be fractionated into a delivery mechanism and the active ingredients (e.g., a pill and the drug compound), so too can digital therapeutics be fractionated into delivery mechanisms and their active ingredients (e.g., screen views and scheduling an in-app behavioral activation). However, identifying these active ingredients can prove difficult, as this fractionated process is still under development for face-to-face interventions.

**Recommendation: Investigate predictors of positive clinical outcomes and map out possible digital analogs**. We recommend taking a theory-driven approach to identifying clinical outcomes and then validating this with product data. Many digital health interventions are digitized versions of face-to-face interventions; therefore, it can be common to examine theoretical predictors of clinical outcomes in face-to-face interventions and then look for digital analogs (i.e., the digital version of what happens face-to-face) of those factors. As noted above, while these theoretical predictors are still debated within face-to-face interventions, they provide a foundation to start hypothesis testing, particularly for new digital therapeutics.

Coming back to our example to select our measure of meaningful engagement, we first investigate what predicts favorable clinical outcomes in face-to-face CBT for adolescents and identify multiple predictors. Next, we map the theoretical indicators to specific, measurable interactions within the digital intervention.

For example, we may first identify the following predictors of positive clinical outcomes in face-to-face CBT:

1. Showing up for weekly appointments
2. Doing assigned homework (e.g., completing mood-activity logs and behavioral activations) (16)
3. Having a positive therapeutic alliance with the therapist
4. Parent involvement (e.g., showing up to session, supporting teen in homework)

Which may correspond to the following digital analogs:

1. Weekly active usage (a generic engagement measure)
2. Completing specific, in-app therapeutic exercises (e.g., logging behavioral activations)
3. A questionnaire that measures therapeutic alliance as part of weekly symptom check-ins
4. Completion of parent assigned tasks, or adolescent and parent reported ratings of parental support in homework

Based on the above, we might choose completing specific in-app exercises, such as behavioral activation logs, as the most promising engagement metric, because it is the in-app action most closely related to the hypothesized active ingredients within behavioral activation therapy. Despite the heterogeneity of engagement metrics used to evaluate digital therapeutics, in the literature, adherence to the recommend usage has been shown to be strong indicator of positive outcomes (17). This behavior (adherence) is also understood to be influenced by a users' developmental stage (e.g., age).

For new digital therapeutic programs or those with limited data, a large part of this challenge is that there is little to no prior evidence of the leading indicators of clinical outcomes within this modality, which may force one to rely on theory alone. For products with existing data, however, exploratory analyses can be conducted to refine the theory-based hypotheses.

## 3. Implement solutions to increase meaningful engagement

### 3a: Hypothesize theory-driven solutions that will drive meaningful engagement in the digital therapeutic

This can be difficult within the new space of digital therapeutics, when prior solutions to increase engagement were largely based on generic metrics of engagement or consumer based products (e.g., retention for retention's sake; (11).

**Recommendation: Identify engagement techniques based on developmental, behavioral, and clinical science theory and research.** To design solutions that improve clinical outcomes by effectively driving meaningful engagement, we advocate for leveraging an understanding of behavioral change techniques. For a focus on adolescent depression, it is further important to leverage behavioral change theory from a developmental lens. There are many well-established techniques to improve user engagement that are beyond the scope of the article– such as usability, visual design, narratives, goal-setting, self-monitoring, professional support, reminders, interactivity, narrative, user control accountability, personalization, social support, digital therapeutic alliance, credibility, and treatment expectancy (11). Here, we specifically focus on an example with a developmental lens (2, 18–20).

Since the early days of behaviorism, researchers have long established that rewards are one of the most effective ways to influence behavior (21, 22). Reward systems within digital therapeutics generally incentivize target behaviors by providing extrinsic rewards, such as badges, points, or level progression (23). Rewards can be provided for the target behavior itself (e.g., going for a run), effort towards the target behavior (e.g., scheduling a run), or approximations to the target behavior (e.g., going for a walk). Rewards are often grouped into a larger category of gamification elements, which are designed to provide extrinsic motivation to engage with the intervention. Though gamification elements can improve engagement in digital health interventions, it's worth noting there is some debate (24, 25), and there are very few studies examining the precise impact of rewards by comparing the same intervention with and without rewards or other gamification elements. There are other components of gamification that can influence users' motivation beyond reward, however, such as motivation by purpose, autonomy, relatedness, or competence (26). For an adolescent focused intervention, there are unique developmental considerations that influence motivation, reward, and punishment (27–29). Those additional aspects of gamification (e.g., autonomy and relatedness) also have particular developmental relevance to adolescents. For example, during adolescence, young people are gaining more autonomy from their parents, exploring their self-identity, and are neurobiologically more sensitive to social rewards, making them more likely to take a riskier (unknown) option for the opportunity to learn, than choose a known reward (30). A successful adolescent-focused reward structure should integrate those considerations. For example, is there a way to offer a menu of tailored reward options, ensure rewards are salient to your target population, provide frequent and different sizes of rewards toward incremental progress, or add an element of choice regarding when they cash in rewards?

### 3b: Tailor engagement technique for maximum impact

Understanding the theory behind behavioral change techniques is necessary but insufficient to drive engagement. The same behavioral change technique can lead to very different effect sizes (31). A major reason for this is that a behavioral change technique can be implemented in a variety of contexts (32). It is critical to tailor the implementation of the technique to the specific characteristics of your users.

**Recommendation**: **Implement user-centered design processes to fine-tune the implementation of the engagement techniques.** User-centered design is an approach that focuses on users and their needs in every step of the design process (33). To this end, we recommend employing a range of techniques for engaging end-users as creative partners in the design process.

**Figure 2:**
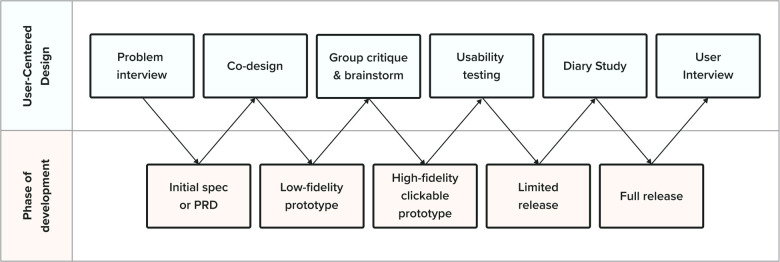
An example product development process paired with steps for user-centered design.

The reason for incorporating a user-centered design process is simple: product developers are not often their users. Due to differences across age, culture, life experience, and cognition, what is engaging to the developer may be different than what is engaging to their users. In building a digital intervention for adolescent depression, even if developers may remember (or think they remember!) what it was like to be an adolescent, there is no substitute for incorporating the voices of end users. Figure 2 illustrates an example product development process paired with steps for user-centered design.

There is a wide range of tools available, and it is essential to know which tool to use at each stage of the product development cycle. The first step of this process is to create a product requirement document (PRD). We can use **problem interviews** at this early stage to identify user needs and how users are currently addressing those needs.

After creating the PRD, we generate initial solution concepts using **co-design.** Co-design involves working with participants to generate potential solutions to a design problem. This often involves a process in which participants sketch out potential solutions, share them with each other, and then iterate [For more details, see (34)].

We then create a low-fidelity prototype, a rudimentary solution abstraction. We gain feedback from users with **group critiques and brainstorming**—sometimes referred to as a solution interview. We brief participants on the design goal, show them early designs, gather feedback, and then ask them to brainstorm improvements as a group.

Next, we create a high-fidelity prototype. We run **usability tests,** which involve users completing defined tasks within a prototype while they think aloud. The goal is to assess how easy the intervention is to use and understand.

After incorporating feedback, the product development team may prepare a limited release for **diary studies**– which involve asking users to document their experiences and thoughts while completing the intervention. Diary studies enable us to get in-the-moment feedback that more accurately represents users' experiences.

After implementing the solution in a larger release (e.g., in a clinical trial), we gather more feedback through **user interviews.** This involves collecting feedback from users after they complete the intervention through a series of structured questions about their experience. Moving from this stage to an even larger public release brings up new challenges and considerations, discussed below.

## 4: Iteratively evaluate the solution's impact on meaningful engagement and clinical outcomes

### 4a: Test product changes

Developing a feature with qualitative feedback (user-centered design process) also requires quantitative evaluation with users to first, evaluate the effect on the meaningful engagement metric and, secondly, to determine if that metric was associated with a change in clinical outcomes. In the wider technology industry, running A/B tests– continuously releasing minor changes to a subset of users and comparing outcomes– is common. However, for digital therapeutics, the impact of this type of testing could have major impacts on users' health and wellbeing, so A/B testing may not be feasible from a safety or regulatory perspective (e.g., if your product is FDA-regulated).

**Recommendation: Run a series of small-scale studies before releasing public changes.** Coming back to our example, we run a series of small-scale Institutional Review Board (IRB)-regulated studies to test specific hypotheses around meaningful engagement. Thus, many digital therapeutic developers build their own internal research infrastructure to run small-scale clinical trials much more quickly and inexpensively than would otherwise be possible by partnering with third-party research organizations. For example, in one study, we can test if breaking down behavioral activations into smaller chunks, with more frequent rewards for incremental completion, increases the number completed (a hypothesized indicator of clinical outcomes).

Once we confirm the feature is safe for adolescents and leads to similar or better clinical outcomes than the existing product version, we can release it to the entire user base.

### 4b: Establishing mechanisms of action

After running each of the above-mentioned trials, it can still be difficult to determine causality and empirically validate whether the hypothesized meaningful engagement metric contributes to clinical outcomes. Psychological processes are complex and require massive data sets to untangle the many competing factors contributing to outcomes. Even in face-to-face interventions, which have undergone decades of research and clinical trials, researchers are still attempting to pinpoint the therapeutic “active ingredients” that contribute to clinical outcomes (35, 36). Furthermore, it is also important to be cautious about mining the data to find correlations [p-hacking or hypothesizing after the fact; (37)], which can lead to spurious conclusions about these mechanisms of action and ultimately irreproducible effects.

**Recommendation: Rely on theory where appropriate and be cognizant of limitations.** There are no easy solutions to this challenge. As digital therapeutics scale, there is real potential to gain the critical mass of data necessary to identify reliable effect sizes. This is one of the major advantages of digital therapeutics over traditional therapies.

In the meantime, we recommend understanding the theoretical mechanisms underlying clinical outcomes and taking a cross-functional approach to triangulating the “why” of an outcome. For example, after we see quantitative support for our hypothesis, we can bring back in user experience research to interview a representative cohort of study participants to better understand the qualitative “why” behind any quantitative patterns we observed. Furthermore, finding a sustained correlation between the use of the leading indicator and the clinical outcomes may be a good indication that we have found a meaningful engagement metric.

Regardless of sample size, to avoid the trap of data mining for correlations that leads to spurious findings, we recommend following transparent and reproducible study design and analysis pipelines (e.g., pre-registration, open code, clearly labeled exploratory findings in studies, and heavier reliance on effect size than *p*-value (38, 39).

## Discussion

We proposed a 4-step framework to tackle common challenges to creating digital therapeutics with meaningful engagement. As the field evolves and more data are available, however, there are additional challenges and opportunities to consider, such as blending multiple metrics, segmentation, measuring behavior outside of the app, and determining the minimum effective dose. For example, there is rarely only one metric of meaningful engagement in an intervention. Instead, there may be multiple metrics, in which case they may be combined into a hybrid measure of engagement or you might categorize someone as meaningfully engaged if they do any two out of a list of five leading indicators within the program in a given week (40, 41). Meaningful engagement is also likely to differ across users. For example, users with more severe symptoms might benefit from a different style of engagement than users with mild-to-moderate symptoms or differ across users of different socioeconomic, geographic, or racial backgrounds. Optimal engagement style may even change for the same user as they progress through the intervention or recovery, or change based on the user’s starting motivation types, as detailed in the Hexard Scale for gamification (26). To this end, SilverCloud and Microsoft recently published an article that outlined their use of machine learning to identify different engagement styles (8). It is also worth noting that constraining meaningful engagement metrics to objective in-app measures may limit the ability to detect real-world clinical outcome improvement. Assessing digital biomarkers (objective and passive user data), such as wearable devices or smartphone interaction patterns may afford a better opportunity to detect real-world indicators of clinical outcomes (42). With the proliferation of digital health apps, standardized frameworks, e.g., the Mobile App Rating Scale (43), will be increasingly useful for evaluating the quality of a mobile app on a number of dimensions, including engagement. To ensure digital therapeutics meet a high quality bar for engagement, it will be prudent to adopt a cross-functional framework grounded in theoretical, user-centered, and rigorous approaches to design and interpretation to optimally determine meaningful engagement, and ultimately improve clinical outcomes for the intended users.
